# Supplementary material for: A Poisson distribution-based general model of cancer rates and a cancer risk-dependent theory of aging
Source: Aging (Albany NY). 2023 Sep 1;15(17):8537–51. doi: 10.18632/aging.205016 (PMC10522393; doi:10.18632/aging.205016)
Supplement: Supplementary Table 1 [file aging-15-205016-s001.pdf]

SUPPLEMENTARY TABLE

Supplementary Table 1. List of species with different lifespan models.

Strategy I	Strategy II	Strategy III
North American beaver	Hippopotamus	Humans (Hadza hunter-gathers)
Soay sheep	Eastern gorilla	Fin whale
Cheetah	Brown bear	African elephant
European badger	Blue monkey	Killer whale
Meerkat	Steller sea lion	Short-finned pilot whale
Raccoon	Polar bear	Chimpanzee
Lechwe	Steller sea lion	
Pyrenean chamois	Hawaiian monk seal	
Arctic fox	Walrus	
Banded mongoose	Olive baboon	
American red squirrel	White-headed capuchin	
Belding's ground squirrel	Yellow baboon	
Golden-mantled ground squirrel	Northern fur seal	
	Australian fur seal	
	Japanese serow	
	Plains zebra	
	American bison	
	Antarctic fur seal	
	Bighorn sheep	
	Himalayan tahr	
	Leopard	
	Moose	
	Red deer	
	Weddell seal	
	Lion	
	Reindeer	
	Ring-tailed lemur	
	Collared peccary	
	Japanese macaque	
	Yellow-bellied marmot	
